# Roxithromycin monotherapy inducing a partial response in a patient with myeloma: a case report

**DOI:** 10.1186/s13256-018-1636-9

**Published:** 2018-05-10

**Authors:** Kern Y. Chai, Anna L. Byrne, Ian M. Morison

**Affiliations:** 1 Department of Pathology, University of Otago, Hercus Building, 56 Hanover St, Dunedin, 9016 New Zealand; 2 Southern Blood & Cancer Service, Dunedin Hospital, 201 Great King St, Dunedin, 9016 New Zealand

**Keywords:** Roxithromycin, Clarithromycin, Myeloma

## Abstract

**Background:**

Clarithromycin is an efficacious treatment for myeloma in combination with other anti-myeloma therapy but not as monotherapy. To date, all studies have focused on a clarithromycin-specific effect rather than a class effect (macrolide) and there is no information on the activity of roxithromycin in myeloma.

**Case presentation:**

Here we report an untreated 86-year-old New Zealand European white man with IgA myeloma whose paraprotein decreased by 57%, consistent with a partial response, after a course of roxithromycin for pneumonia. His paraprotein reduced from 46 to 20 g/L while his hemoglobin improved from 97 to 123 g/L after 1 month.

**Conclusion:**

Additional investigations should be considered to elucidate the therapeutic effect of roxithromycin in myeloma.

## Background

The addition of clarithromycin, a macrolide antibiotic, to immunomodulatory agents has been shown to improve the efficacy of treatment of patients with newly diagnosed and relapsed refractory myeloma. For example, in 2002, Coleman and colleagues first showed that the addition of clarithromycin to thalidomide in combination with dexamethasone improved the overall response rate [1, 2]. Subsequently, Niesvizky and colleagues reported that in 72 patients with newly diagnosed myeloma, the combination of clarithromycin, lenalidomide, and dexamethasone (BiRD), led to a response rate of 90% with 39% achieving at least a complete remission [2]. To date, all studies have focused on a clarithromycin-specific effect rather than a class effect (macrolide), and there is no information on the activity of roxithromycin in myeloma. We report a case of a patient with IgA myeloma whose paraprotein halved after a course of roxithromycin for a community acquired pneumonia.

## Case presentation

**Fig. 1:**
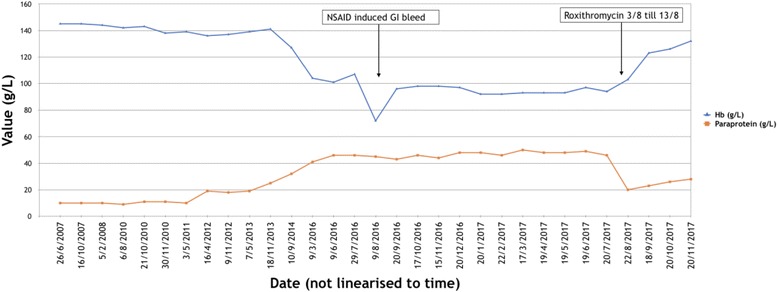
Line graph of paraprotein and hemoglobin since diagnosis. *GI* gastrointestinal, *Hb* hemoglobin, *NSAID* non-steroidal anti-inflammatory drug

An 86-year-old New Zealand European white man was diagnosed as having smoldering myeloma of IgA lambda subtype in June 2008. His diagnostic bone marrow aspirate showed 38% plasma cells. His paraprotein at diagnosis was 9 g/L and gradually increased to 46 g/L by mid-2016 without any evidence of end organ damage (Fig. 1).

His other medical history includes L2–3, L5–S1 spondylosis and early stage prostate adenocarcinoma, T2A Nx M0, Gleason grade 6, which was successfully eradicated with radical radiotherapy in 2000. He takes cholecalciferol 1.25 mg once monthly, cilazapril 2.5 mg daily, amitriptyline 10 mg daily, and paracetamol 1 g twice daily and is intolerant of Augmentin (amoxicillin-potassium clavulanate; diarrhea). He lives with his wife and is fully independent with all his activities of daily living. He does not smoke tobacco and he consumes two units of alcohol per day.

In June 2016, he developed mild normochromic anemia of 104 g/L and 6 weeks later he was hospitalized with melena in the context of recent non-steroidal anti-inflammatory drug (NSAID) use for low back pain. No bleeding source was identified on upper and lower endoscopies. The ibuprofen was stopped and he was started on omeprazole. He was also found to be mildly vitamin B12 deficient with a raised methylmalonic acid, and was commenced on vitamin B12 treatment. His hemoglobin fell to a nadir of 72 g/L during the NSAID-induced gastrointestinal bleed, but quickly recovered to its new baseline of 92–97 g/L and remained unchanged for the next 12 months. With regards to his back pain, a computed tomography (CT) skeletal survey and subsequent magnetic resonance imaging (MRI) of his lumbar spine showed mild osteoporotic compression fractures of L1–4 without any evidence of lytic disease. A follow-up bone density scan confirmed osteoporosis with a T-score of −2.7 in the left neck of his femur. He was treated with a single dose of zoledronic acid in August 2016 and a weaning course of analgesia. Concordant with his hemoglobin, his paraprotein remained stable at 45–50 g/L over the subsequent 12 months. Given the absence of other myeloma-defining events and unclear etiology of the anemia, he continued with watchful observation.

In August 2017, he was prescribed a 10-day course of roxithromycin 150 mg twice daily for a clinically diagnosed community-acquired pneumonia. He had presented to his general practitioner with a 10-day history of productive cough, malaise, and anorexia. His temperature was 37.4 °C, blood pressure 140/70 mmHg, heart rate 70 beats per minute, and O_2_ saturations 98% on room air. Focal crepitations were heard at his left base and the rest of his physical examination was unremarkable. On review 4 days later, he had clinically deteriorated with a temperature of 39.0 °C, blood pressure 140/70 mmHg, heart rate 100 beats per minute, and O_2_ saturations 97% on room air but his physical examination findings were otherwise unchanged. Cephalexin 500 mg twice daily for 1 week was added to his treatment regime. He completed full courses of both antibiotics with eventual complete recovery. No blood test or sputum sample was submitted to the laboratory, and a chest X-ray was not performed. He did not receive any corticosteroids or herbal remedies with his chest infection.

At his next routine hematology follow-up a week later, his paraprotein level (quantified by capillary electrophoresis) had decreased by 57% from 46 to 20 g/L. Over the next 4 months, while off all treatment, it slowly increased to 23, 26 then 28 g/L. Quantitative IgA levels demonstrated a similar pattern, falling from 54 to 22 g/L, before increasing to 27, 31 then 33 g/L. His hemoglobin increased from a pre-treatment average of 97 to 123, 126 then 132 g/L over the next 4 months. The rest of his laboratory parameters remained stable; his creatinine was 102 mmol/L, adjusted calcium 2.3 mmol/L, and albumin 32 g/L. Serum free light chains were not measured. When reviewed 4 months after the course of roxithromycin, he was asymptomatic apart from his chronic intermittent low back pain, which was improving.

His International Staging System was calculated as II in August 2016. Clearly, his myeloma had a favorable slow progressive phenotype, as evidenced by the prolonged latent phase (8 years) from diagnosis and the stability of symptoms and paraprotein despite being untreated.

## Discussion

In summary, our patient is an 86-year-old man with IgA myeloma who was previously “labelled” as asymptomatic and, hence, not treated. His paraprotein showed an impressive 57% reduction, consistent with the 2016 International Myeloma Working Group definition of a partial response [3], following 10 days of roxithromycin and 7 days of cephalexin for a community acquired pneumonia. Cephalexin is not known to have any direct anti-cancer activity whereas the anti-myeloma effect of clarithromycin is well studied [1, 2, 4–7]. There are no reports on the effect of roxithromycin in myeloma. We hypothesize that in the absence of other interventions, roxithromycin is the most likely cause of the impressive reduction in paraprotein level. This case report is unique because it is the first recognition that roxithromycin probably has significant anti-myeloma activity.

Roxithromycin belongs to a family of broad spectrum macrolide antibiotics which are characterized by a macrocyclic lactone ring [8]^.^ They are commonly used to treat bacterial infections including atypical pneumonias and *Helicobacter pylori*. A closely related macrolide antibiotic, clarithromycin, has been a compound of interest in treating patients with myeloma since 1997 when Durie and colleagues found that 13/23 patients with relapsed myeloma achieved a partial response or better [9]. However, that study was confounded by concurrent use of other anti-myeloma therapy (dexamethasone/pamidronate) [4]. Clarithromycin then fell out of favor as three further studies examining clarithromycin as a single agent in the following three years reported no activity [10–12].

Although the clarithromycin monotherapy trials were disappointing, clarithromycin was later shown to have a synergistic effect in combination with other anti-myeloma drugs. Phase 2 trials showed the addition of clarithromycin to thalidomide/dexamethasone improved response rates (> 50% reduction in monoclonal protein level) [1, 2], whereas the combination with lenalidomide/dexamethasone (RD) tripled the complete remission rates from 13.9 to 45.8% [5]. Later, Ghosh and colleagues showed 48% of patients previously resistant to immunomodulatory agents achieved a hematological response after the addition of clarithromycin [6]. Given clarithromycin is a well-known inhibitor of the cytochrome P450 3A4 (CYP3A4) and P-glycoprotein metabolic pathway, the observed effect was thought to reflect the potentiation of corticosteroid activity. The first phase 3 randomized controlled trial commenced in August 2015, comparing BiRD versus RD in newly diagnosed patients, but the preliminary results had not been published at the time of writing (NCT02516696).

The first proof of direct anti-tumor activity of clarithromycin in myeloma cells was shown by Nakamura and colleagues who demonstrated that it blocked autophagy in a dose-dependent manner [13]. Qiu and colleagues subsequently showed that clarithromycin reduced interleukin-6 (IL-6) and tumor necrosis factor (TNF) activity in myeloma cell lines [14]. Both cytokines are key mediators for myeloma cell proliferation, survival, migration, and drug resistance.

This is the first case report on roxithromycin use in myeloma. Similar to its cousin compound, roxithromycin has been shown to reduce the secretion of proinflammatory cytokines including IL-6 and TNF *in vitro,* albeit in respiratory epithelial cells [15]. Of interest, roxithromycin has also been shown to inhibit, in mouse hepatocellular carcinoma models, activation of the nuclear factor kappa-light-chain-enhancer of activated B cells (NFkB) pathway, which is aberrantly up-regulated in myeloma [16].

In contrast to the low efficacy of clarithromycin as monotherapy, this case report suggests roxithromycin could have significant single-agent anti-myeloma activity. A plausible explanation lies in the different pharmacokinetic profiles of these two medications. Roxithromycin has higher bioavailability and half-life, which translates to substantially higher serum and tissue concentrations than the equivalent dose of clarithromycin [8].

## Conclusion

Given the low toxicity and cost of roxithromycin, additional investigation of its therapeutic effects should be considered and it would be a welcome addition to anti-myeloma therapy, especially in countries with limited access to expensive novel therapies.

## Abbreviations

BiRD: Clarithromycin/lenalidomide/dexamethasone
CT: Computed tomography
CYP3A4: Cytochrome P450 3A4
IL-6: Interleukin-6
MRI: Magnetic resonance imaging
NFkB: Nuclear factor kappa-light-chain-enhancer of activated B cells
NSAID: Non-steroidal anti-inflammatory drug
RD: Lenalidomide/dexamethasone
TNF: Tumor necrosis factor

## Authors’ information

IM: Professor of Pathology, University of Otago.

KC: Hematology advanced trainee (RACP & RCPA), Dunedin Hospital.

AB: Consultant hematologist, Dunedin Hospital.

## References

[1] Coleman M, Leonard J, Lyons L, Pekle K, Nahum K, Pearse R, et al. BLT-D (Clarithromycin [Biaxin], Low-Dose Thalidomide, and Dexamethasone) for the Treatment of Myeloma and Waldenström’s Macroglobulinemia. Leuk Lymphoma. 2002;43:1777–82. 10.1080/1042819021000006303 12685831

[2] Niesvizky R, Jayabalan DS, Christos PJ, Furst JR, Naib T, Ely S, et al. BiRD (Biaxin [clarithromycin]/Revlimid [lenalidomide]/dexamethasone) combination therapy results in high complete- and overall-response rates in treatment-naive symptomatic multiple myeloma. Blood. 2008;111:1101–9. 10.1182/blood-2007-05-090258 17989313

[3] Kumar S, Paiva B, Anderson KC, Durie B, Landgren O, Moreau P, et al. International Myeloma Working Group consensus criteria for response and minimal residual disease assessment in multiple myeloma. Lancet Oncol. 2016;17:e328–46. 10.1016/S1470-2045(16)30206-6 27511158

[4] Van Nuffel AM. Repurposing Drugs in Oncology (ReDO)—clarithromycin as an anti-cancer agent. Ecancermedicalscience. 2015;9:1–26. 10.3332/ecancer.2015.513 PMC4341996 25729426

[5] Gay F, Rajkumar SV, Coleman M, Kumar S, Mark T, Dispenzieri A, et al. Clarithromycin (Biaxin)-lenalidomide-low-dose dexamethasone (BiRd) versus lenalidomide-low-dose dexamethasone (Rd) for newly diagnosed myeloma. Am J Hematol. 2010;85:664–9. 10.1002/ajh.21777 PMC3956597 20645430

[6] Ghosh N, Tucker N, Zahurak M, Wozney J, Borrello I, Huff CA. Clarithromycin overcomes resistance to lenalidomide and dexamethasone in multiple myeloma. Am J Hematol. 2014;89:E116–20. 10.1002/ajh.23733 PMC4390045 24723438

[7] Kato H, Onishi Y, Okitsu Y, Katsuoka Y, Fujiwara T, Fukuhara N, et al. Addition of clarithromycin to lenalidomide/low-dose dexamethasone was effective in a case of relapsed myeloma after long-term use of lenalidomide. Ann Hematol. 2013;92:1711–2. 10.1007/s00277-013-1761-x 23625297

[8] Bahal N, Nahata MC. The New Macrolide Antibiotics: Azithromycin, Clarithromycin, Dirithromycin, and Roxithromycin. Ann Pharmacother. 2016;26:46–55. 10.1177/106002809202600112 1318761

[9] Durie BGM, Villarete L, Farvard A, Ornopia M, Urnovitz HB. Clarithromycin (Biaxin) as primary treatment for myeloma. Blood. 1997:90(suppl 1):2578.

[10] Stewart AK, Trudel S, Al-Berouti BM, Sutton DM, Meharchand J. Lack of response to short-term use of clarithromycin (BIAXIN) in multiple myeloma. Blood. 1999;93:4441. 10391696

[11] Moreau P, Huynh A, Facon T, Bouilly I, Sotto JJ, Legros L, et al. Lack of efficacy of clarithromycin in advanced multiple myeloma. Leukemia. 1999;13:490–1. 10.1038/sj.leu.2401332 10086745

[12] Musto P, Falcone A, Sanpaolo G, Bodenizza C, Carotenuto M, Carella AM. Inefficacy of clarithromycin in advanced multiple myeloma: a definitive report. Haematologica. 2002;87:658–9. 12031924

[13] Nakamura M, Kikukawa Y, Takeya M, Mitsuya H, Hata H. Clarithromycin attenuates autophagy in myeloma cells. Int J Oncol. 2010;37:815–20. 20811702

[14] Qiu X-H, Shao J-J, Mei J-G, Li H-Q, Cao H-Q. Clarithromycin Synergistically Enhances Thalidomide Cytotoxicity in Myeloma Cells. Acta Haematol. 2016;135:103–9. 10.1159/000438855 26505646

[15] Gao X, Ray R, Xiao Y, Barker PE, Ray P. Inhibition of sulfur mustard-induced cytotoxicity and inflammation by the macrolide antibiotic roxithromycin in human respiratory epithelial cells. BMC Cell Biol. 2007;8:17. 10.1186/1471-2121-8-17 PMC1890552 17524151

[16] Ueno S, Aoki D, Kubo F, Hiwatashi K, Matsushita K, Oyama T, et al. Roxithromycin inhibits constitutive activation of nuclear factor κB by diminishing oxidative stress in a rat model of hepatocellular carcinoma. Clin Cancer Res. 2005;11:5645–50. 10.1158/1078-0432.CCR-05-0652 16061884

